# Differential effects of ELX/TEZ/IVA on organ-specific CFTR function in two patients with the rare CFTR splice mutations c.273+1G>A and c.165-2A>G

**DOI:** 10.3389/fphar.2023.1153656

**Published:** 2023-03-27

**Authors:** Sophia T. Pallenberg, Inka Held, Christian Dopfer, Rebecca Minso, Manuel M. Nietert, Gesine Hansen, Burkhard Tümmler, Anna-Maria Dittrich

**Affiliations:** ^1^ Department of Pediatric Pneumology, Allergology and Neonatology, Hannover Medical School, Hannover, Germany; ^2^ German Center for Lung Research, Biomedical Research in Endstage and Obstructive Lung Disease (BREATH), Hannover Medical School, Hannover, Germany; ^3^ Kinderärzte Friesenweg—CF-Zentrum Altona (Ambulanz), Hamburg, Germany; ^4^ Department of Medical Bioinformatics, University Medical Center Göttingen, Göttingen, Germany

**Keywords:** cystic fibrosis transmembrane conductance regulator (CFTR), elexacaftor/tezacaftor/ivacaftor, c.165-2A>G, CFTR rare mutations, exon 3

## Abstract

**Introduction:** Evidence for the efficiency of highly-effective triple-CFTR-modulatory therapy with elexacaftor/tezacaftor/ivacaftor (ETI), either demonstrated in clinical trials or by *in vitro* testing, is lacking for about 10% of people with cystic fibrosis (pwCF) with rare mutations. Comprehensive assessment of CFTR function can provide critical information on the impact of ETI on CFTR function gains for such rare mutations, lending argument of the prescription of ETI. The mutation c.165-2A>G is a rare acceptor splice mutation that has not yet been functionally characterized. We here describe the functional changes induced by ETI in two brothers who are compound heterozygous for the splice mutations c.273+1G>C and c.165-2A>G.

**Methods:** We assessed the effects of ETI on CFTR function by quantitative pilocarpine iontophoresis (QPIT), nasal potential difference measurements (nPD), intestinal current measurements (ICM), β-adrenergic sweat secretion tests (SST) and multiple breath washout (MBW) prior to and 4 months after the initiation of ETI.

**Results:** Functional CFTR analysis prior to ETI showed no CFTR function in the respiratory and intestinal epithelia and in the sweat gland reabsorptive duct in either brother. In contrast, β-adrenergic stimulated, CFTR-mediated sweat secretion was detectable in the CF range. Under ETI, both brothers continued to exhibit high sweat chloride concentration in QPIT, evidence of low residual CFTR function in the respiratory epithelia, but normalized β-adrenergically stimulated production of primary sweat.

**Discussion:** Our results are the first to demonstrate that the c.165-2A>G/c.273+1G>C mutation genotype permits mutant CFTR protein expression. We showed organ-specific differences in the expression of CFTR and consecutive responses to ETI of the c.165-2A>G/c.273+1G>C CFTR mutants that are probably accomplished by non-canonical CFTR mRNA isoforms. This showcase tells us that the individual response of rare *CFTR* mutations to highly-effective CFTR modulation cannot be predicted from assays in standard cell cultures, but requires the personalized multi-organ assessment by CFTR biomarkers.

## 1 Introduction

Cystic fibrosis (CF) is a severe ion channel disease of autosomal recessive inheritance that is caused by mutations in the *Cystic Fibrosis Transmembrane Conductance Regulator (CFTR)* gene (Stoltz et al., 2015; Shteinberg et al., 2021). CFTR is a low conductance anion-selective, ATP-regulated ion channel. Its major role is to regulate chloride and bicarbonate ion movements across epithelial tissues throughout the body (Stoltz et al., 2015). More than 2,000 mutations and polymorphisms are known in the *CFTR* gene, with rare mutations remaining to be functionally characterized (Tümmler et al., 1996).

CF is the first successful example of customized drug development for mutation-specific therapy (Tümmler, 2022). CFTR correctors have been developed for improved posttranslational maturation and trafficking of mutants such as p.Phe508del that do not achieve a stable fully-folded polytopic configuration. Potentiators of CFTR activity increase chloride and bicarbonate flux across apical epithelial membranes. The potentiator Ivacaftor enhances the ATP-independent opening of the CFTR channel and thereby overcomes the defective ATP-dependent opening of CF-causing gating mutations (Van Goor et al., 2009; Eckford et al., 2012). The new triple combination therapy with the correctors elexacaftor and tezacaftor and the potentiator ivacaftor has shown to be highly efficient for the large group of patients with one or two p.Phe508del alleles regarding lung function and reduction of sweat chloride concentration (Middleton et al., 2019; Barry et al., 2021; Zemanick et al., 2021; Graeber et al., 2022; Sutharsan et al., 2022) and improvement of CFTR function in airway and intestinal epithelia (Graeber et al., 2022).

To date, approximately 90% of people with cystic fibrosis (pwCF) are eligible for highly-effective triple-CFTR-modulatory therapy with elexacaftor/tezacaftor/ivacaftor (ETI) as demonstrated in clinical trials or *in vitro* testing. However, such evidence is lacking for about 10% of pwCF with rare mutations where clinical trials are unlikely to address their response to ETI. In these cases, comprehensive assessment of CFTR function can provide critical information on the impact of ETI on CFTR function gains for such rare mutations, lending argument of the prescription of ETI.

We here describe assessment of organ-specific *in vivo* and *ex vivo* baseline CFTR function and the functional changes induced by ETI in two brothers (B1 and B2) who are compound heterozygous for the splice mutations c.273+1G>C and c.165-2A>G. c.273+1G>A is a donor splice mutation at position c.273+1 of the first nucleotide in intron 3 of the *CFTR* gene, where the obligatory conserved guanine (G) has been replaced by an adenine (A) (Tümmler, 2022). This leads to the absence of exon 3 in the CFTR mRNA messenger and a reading frame shift of the CFTR mRNA, causing different amino acids to be encoded from exon 4 onward and repeated stop signaling (Dörk et al., 1993). In other words, the c.273+1G>A mutation is a “loss of function” mutation, causing the inability to synthesize a functional CFTR protein. The mutation is assigned to *CFTR* mutation class 1.

In the acceptor splice mutation c.165-2A>G, adenine (A) is exchanged for guanine (G) at the penultimate position in intron 2. The mutation is rare and has not yet been characterized in terms of its effects on CFTR mRNA composition. However, based on CFTR acceptor splice mutations in other introns of the CFTR gene, we can again expect the skipping of exon 3 as the major consequence of the c.165-2 A>G mutation. Like c.273+1G>A, c.165-2 A>G should be a class I mutation.

The two mutations affect the canonical splice sites flanking exon 3. Exon 3 skipping from both alleles should lead to a knock-out phenotype of no functional CFTR protein provided that no other CFTR mRNA isoforms are produced. However, as shown in this report, an organ-specific rescue of CFTR function was observed by CFTR biomarkers in the two index cases who are compound heterozygous for the rare splice mutations c.165-2A>G and c.273+1G>C. The two brothers showed residual CFTR activity in sweat secretion and gained more CFTR activity during ETI triple therapy in the respiratory epithelium and the secretory coil of the sweat gland.

## 2 Materials and methods

In this study, we assessed the effects of ETI on CFTR function in two brothers (B1 and B2) who are compound heterozygous for the splice mutations c.273+1G>C and c.165-2A>G. For clinical evaluation, we monitored the lung clearance index 2.5 (LCI_2.5_) by multiple breath washout (MBW). We quantified CFTR-function using quantitative pilocarpine iontophoresis (QPIT), nasal potential difference measurements (nPD), intestinal current measurements (ICM) and β-adrenergic sweat secretion tests (SST). All tests were performed prior to and 4 months after the initiation of ETI.

MBW testing was performed with the Exhalyzer D system (Eco Medics), and 100% oxygen was used to wash out resident nitrogen from the lungs with a mouthpiece as interface (Stahl et al., 2017). All measurements were using spiroware 3.3.1 (Eco Medics) (Stahl et al., 2020; Graeber et al., 2021; Wyler et al., 2021). The upper limit of normal (ULN) was determined as 7.1 (Wyler et al., 2021).

QPIT was performed following the German national diagnostic guideline (Nährlich et al., 2013) and the guidelines of the Clinical and Laboratory Standards Institute (Wayne, 2009). Pilocarpine iontophoresis was used to stimulate the skin and sweat was collected with the Macroduct^®^ system (Model 3700, Wescor, Logan UT, United States). Sweat chloride concentration was measured using a chloridometer (KWM 20 Chloridometer, Kreienbaum, Langenfeld, Germany) in a minimum volume of 30 μL.

NPD measurements were performed according to the Standard Operating Procedure nPD_EU001, version 1.7 (March 2013) “Nasal Potential Difference (nPD) Measurement for Diagnosis and Clinical Trials in Cystic Fibrosis” of the European Cystic Fibrosis Society (ECFS) Diagnostic Network Working Group and Clinical Trials Network and as previously described (Sermet-Gaudelus et al., 2010; Rowe et al., 2011; Graeber et al., 2018) and published recently (Graeber et al., 2022). The Sermet Score was used to discriminate between normal (>0.27) and reduced (<0.27) CFTR function in the respiratory epithelium of the nose (Sermet-Gaudelus et al., 2010).

ICM was performed according to the Standard Operating Procedure ICM_EU001, version 2.7 (October 2011) “Ion Transport in Rectal Biopsies for Diagnosis and Clinical Trials in Cystic Fibrosis” of the European Cystic Fibrosis Society (ECFS) Diagnostic Network Working Group and Clinical Trials Network modified by in-house protocol adjustments at the CF electrophysiology laboratory in Hannover (Graeber et al., 2015; Graeber et al., 2018). CFTR function in rectal tissue biopsies was quantified using the response to forskolin/IBMX and carbachol in μA/cm^2^.

The β-adrenergic sweat secretion test was performed as previously published (Pallenberg et al., 2022) using the AutoBuSTeD software for automatic analysis of sweat bubble formation. Sweat rates were measured in sweat volume (nL) per time (min). A β-adrenergic sweat rate of < 0.16 nL/min was defined as impaired CFTR function.

### 2.1 Statistical analysis

We analyzed all data with GraphPad Prism version 9.0.1 (GraphPad Software) and R 3.6.2 (R Core Team, 2018).

## 3 Results

### 3.1 Clinical characteristics

**FIGURE 1:**
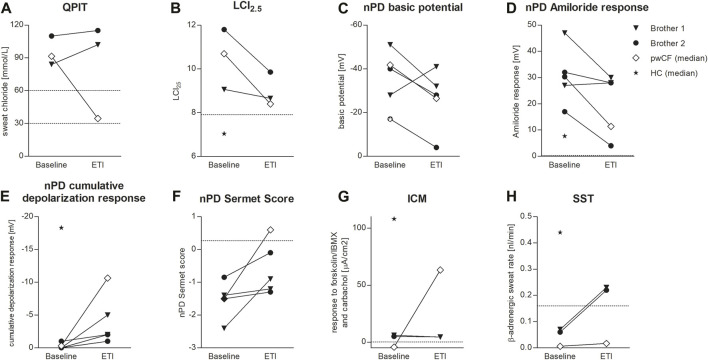
Effects of ETI on QPIT **(A)**, LCI_2.5_ **(B)**, nPD basic potential **(C)**, nPD Amiloride response **(D)**, nPD cumulative depolarization response to chloride-free solution and isoproterenol **(E)**, nPD Sermet Score **(F)**, response to forskolin/IBMX and carbachol in ICM **(G)** and SST **(H)** in brother 1 (triangle) and brother 2 (circle) compared to the median effect on pwCF with one or two p.Phe508del alleles (white square) and reference values for healthy controls (HC, star). Dashed lines indicate published limits of normal for QPIT (Nährlich et al., 2013), LCI_2.5_ (Wyler et al., 2021), nPD Sermet Score (Sermet-Gaudelus et al., 2010) and SST (Pallenberg et al., 2022).

Both patients were compound heterozygous for the rare splice mutations c.273+1G>C and c.165-2A>G. At baseline, B1 was 10.2 years old and B2 was 7.2 years, both of them presented with the phenotype of pancreatic insufficient cystic fibrosis (PI-CF). Prior to ETI, the sweat chloride concentrations were in the typical PI-CF range (B1: 84 mmol/L, B2: 110 mmol/L) and LCI_2.5_ values showed moderate lung ventilation inhomogeneity (B1: 9.06, B2: 11.8). After 4 months of CFTR modulator therapy with ETI, sweat chloride concentrations remained in the PI-CF range (B1: 102 mmol/L, B2: 115 mmol/L, Figure 1A) and only modest improvements in pulmonary function were seen in the LCI_2.5_ values (B1: 8.66, B2: 9.86, Figure 1B; Table 1).

**TABLE 1:** Clinical and functional parameters of the two brothers before and under ETI therapy and reference values for pwCF with one (∆/MF) or two (∆/∆) p.Phe508del alleles without CFTR-modulator therapy (baseline) and after 3 months of ETI (ETI) and healthy controls (HC).

**Clinical parameters**	**Brother 1 (B1)**				**Brother 2 (B2)**				**References values**				
	**Baseline**		**ETI**		**Baseline**		**ETI**		**pwCF (baseline) median**		**pwCF (ETI) median**		**HC median**
									**∆/MF**	**∆/∆**	**∆/MF**	**∆/∆**	
Gender	m				m				—		—		—
Phenotype	PI-CF				PI-CF				—		—		—
Age in years	10.2		10.5		7.2		7.5		—		—		—
LCI_2.5_	9.06		8.66		11.8		9.86		10.3^a^	10.7^a^	7.4^a^	8.4^a^	<7.1 (ULN)^b^
Sweat chloride [mmol/L]	84		102		110		115		103^a^	91.5^a^	50^a^	34.5^a^	<30^c^
**nPD**	*Right*	*Left*	*Right*	*Left*	*Right*	*Left*	*Right*	*Left*					
Basic potential [mV]	−51	−28	−32	−41	−17	−40	−4	−28	−42.2^d^	−41.7^d^	−28^d^	−26.4^d^	−16.8^e^
Amiloride response [mV]	47	27	30	28	17	32	4	28	24.8^d^	30.3^d^	15.1^d^	11.4^d^	7.7^e^
Cumulative depolarization response to chloride-free solution and isoproterenol [mV]	0	0	−5	−2	0	−1	−1	−2	−1.1^d^	−0.3^d^	−9.9^d^	−10.6^d^	−18.3^e^
Sermet Score	−2.4	−1.4	−0.9	−1.2	−0.85	−1.5	−0.1	−1.3	−1.1^d^	−1.5^d^	0.4^d^	0.6^d^	> 0.27^e^
**ICM**													
Response to forskolin/IBMX and carbachol [μA/cm^2^]	6		4.4		5		4.5		−1.9^d^	−4.4^d^	59.1^d^	63.3^d^	108^f^
**SST**													
β-adrenergic sweat rate [nL/min]	0.07		0.23		0.06		0.22		0.006^g^		0.017^g^		0.44^g^

^a^ (Graeber et al., 2022).

^b^ (Wyler et al., 2021).

^c^ (De Boeck et al., 2006).

^d^ (Graeber et al., 2022).

^e^ (Sermet-Gaudelus et al., 2010).

^f^ (Minso et al., 2020).

^g^ (Pallenberg et al., 2022).

### 3.2 Effects of ETI on the CFTR function of the respiratory epithelia

CFTR function in the respiratory epithelium was determined by nasal potential difference measurement. At baseline, B1 presented with typical findings for PI-CF (for reference values see Table 1). The basic potential was in the CF range (right nostril: −51 mV, left nostril: −28 mV, Figure 1C) and we saw a high hyperpolarization response to amiloride (right: 47 mV, left: 27 mV, Figure 1D). The cumulative depolarization response to chloride-free solution and isoproterenol was absent (left and right nostril: 0 mV, Figure 1E). Under ETI, there were no relevant changes in basic potential or amiloride response. However, cumulative depolarization response to chloride-free solution and isoproterenol improved slightly as a sign of low residual CFTR function under ETI (right nostril: −5 mV, left nostril −2 mV). The Sermet score improved to some extent but remained in the CF-range (<0.27) from −2.4 to −0.9 (right nostril) and −1.4 to −1.2 (left nostril; Figure 1F; Table 1).

Prior to ETI, B2 showed a normal basic potential in the right nostril (−17 mV, Figure 1C), hyperpolarization to amiloride in the borderline CF range (17 mV, Figure 1D), and a cumulative depolarization response to chloride-free solution and isoproterenol in the CF range (0 mV, Figure 1E). In the left nostril, all values were in the typical CF range. Four months of ETI therapy led to no significant improvements in CFTR function as measured by cumulative depolarization response (Figure 1E). The basic potential increased from −17 to −4 mV (right nostril) and −40 to −28 mV (left nostril, Figure 1C). At baseline, the Sermet Score was −0.85 (right nostril) and −1.5 (left nostril) and showed mild improvements to −0.1 (right nostril) and −1.3 (left nostril) under ETI, remaining in the CF range (<0.27) (Figure 1F; Table 1).

### 3.3 Measurement of intestinal CFTR function in rectal biopsies

In intestinal current measurement (ICM), CFTR function in rectal tissue biopsies was quantified by the response to forskolin/IBMX and carbachol in μA/cm^2^. Prior to ETI, stimulation with forskolin/IBMX and carbachol induced cumulative ion currents of 6 μA/cm^2^ in the biopsies of B1 and 5 μA/cm^2^ in the biopsies of B2. Compared to our data from 68 healthy controls with a median response to forskolin/IBMX and carbachol of 108 μA/cm^2^ (IQR: 63–198 μA/cm^2^, Minso et al., 2020) and our data from pwCF with one or two p.Phe508del alleles with a median response of −1.9 and −4.4 µA/cm^2^ (Graeber et al., 2022; Table 1), the brothers presented typical responses in the CF range. Thus, there was no evidence of CFTR-mediated chloride secretion in the intestinal epithelium at baseline. After 4 months of therapy with ETI, stimulation with forskolin/IBMX and carbachol induced cumulative ionic currents of 4.4 μA/cm^2^ in the biopsies of B1 and 4.5 µA/cm^2^ in the biopsies of B2, concluding in no improvement of CFTR-function in the intestinal epithelium (Figure 1G).

### 3.4 Effect of ETI on the β-adrenergic sweat rate

**FIGURE 2:**
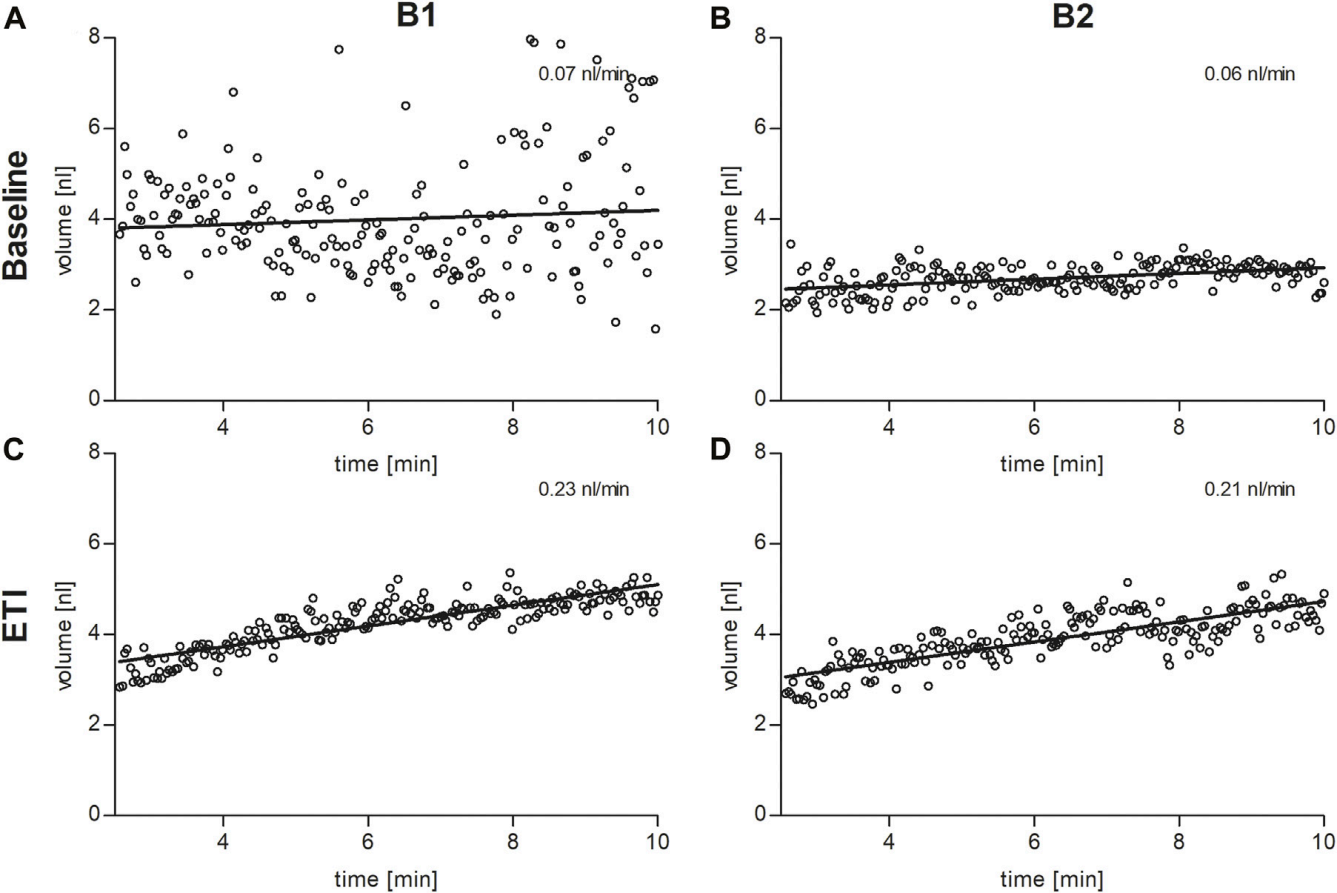
Sweat bubble formation after β-adrenergic stimulation of the skin at baseline **(A,B)** and under ETI **(C,D)** in brother 1 (B1, left column) and brother 2 (B2, right column). Each dot represents the median bubble volume per time point as calculated by the AutoBuSTeD software (Pallenberg et al., 2022). The line shows the linear correlation, the slope corresponds to the sweat rate in nL/min.

In the secretory epithelium of the sweat gland, following β-adrenergic stimulation with concomitant cholinergic inhibition by atropine, CFTR-mediated sweat secretion is detected as a direct indicator of CFTR function (Sato and Sato, 1984; Wine, 2022). In contrast, determination of chloride concentration in the sweat indicates the capacity for CFTR-mediated chloride reabsorption in the excretory duct of the sweat gland. We used the AutoBuSTeD software for automated processing of sweat bubble formation and our previously published protocol to analyze sweat rates in nL/min (Figure 2). Our reference values for pwCF with one or two p.Phe508del alleles (median 0.006 nL/min; IQR 0–0.027 nL/min) and healthy controls (median 0.44 nL/min; IQR 0.34–0.48 nL/min) determined the CF range (Table 1). A cut-off value as calculated by ROC analysis between these two reference cohorts of 0.16 nL/min was used to discriminate between normal and impaired CFTR function (Pallenberg et al., 2022). Prior to ETI, both brothers showed a β-adrenergic sweat rate in the upper CF range (B1: 0.07 nL/min, B2: 0.06 nL/min), indicating impaired but residual CFTR function in the sweat gland. Therapy with ETI led to a significant increase and normalization of β-adrenergic sweat rates to 0.23 nL/min (B1) and 0.22 nL/min (B2) (Figures 1H, 2).

## 4 Discussion

This case of the two brothers with the rare and, in part, previously uncharacterized CFTR splice mutations showed a divergence of responses in CFTR biomarkers to ETI which, particularly in the sweat gland, was antagonistic to the findings of pwCF with one or two p.Phe508del alleles. Functional CFTR analysis prior to ETI showed no CFTR function in the respiratory (nPD) and intestinal (ICM) epithelia and in the sweat gland reabsorptive duct (QPIT) in either brother. In contrast, β-adrenergic stimulated, CFTR-mediated sweat secretion (SST) was detectable in the CF range. Under ETI, both brothers continued to exhibit high sweat chloride concentration in QPIT, a low total chloride response in ICM, evidence of low residual CFTR function in the respiratory epithelia (nPD), but normalized β-adrenergically stimulated production of primary sweat.

Our results indicate organ-specific differences in the expression of CFTR and consecutive responses to ETI of the c.165-2A>G/c.273+1G>C *CFTR* genotype. Prior to ETI, functional CFTR was detectable only in the secretory epithelium of the sweat gland by SST. ETI normalized CFTR function in the secretory apparatus of the sweat gland, whereas CFTR function in the sweat duct as assessed by QPIT and respiratory epithelium improved poorly or not at all.

The c.165-2A>G mutation affects the canonical splice acceptor site preceding exon 3 and the c.273+1 mutation affects the canonical splice donor site following exon 3. Thus, both mutations are expected to induce exon 3 skipping as the major consequence as it has been demonstrated for c.273+1G>C in the respiratory epithelium of a c.273+1G>C/p.Phe508del compound heterozygous individual with CF (Dörk et al., 1993). However, the c.165-2A>G/c.273+1G>C compound heterozygous brothers showed subtle residual CFTR function in the respiratory epithelium and substantial residual CFTR function in the secretory coil. Since β-adrenergically stimulated chloride secretion in the secretory coil of the sweat gland is exclusively executed by CFTR and is not substituted by any other ion channel (Wine, 2022), we can conclude that chloride secretion was mediated by one or more CFTR mRNA isoforms that confer residual CFTR activity. The use of cryptic splice sites may lead to a non-canonical CFTR mRNA isoform. A cryptic exon is known in intron 3 flanked by almost perfect acceptor and donor splice sites, but it encodes two termination codons so that no functional CFTR activity can be expected from this CFTR mRNA isoform (Will et al., 1994). Alternatively, minute amounts of full-length CFTR mRNA transcript may be produced if the splice mutations are somewhat leaky. This scenario may not sound plausible by first glance. However, exon skipping as the only consequence of canonical splice site mutations flanking exon 3 has been observed in compound heterozygous patients who carry a class II or a class IV missense mutation in trans (Dörk et al., 1993; Bienvenu et al., 1994). In contrast, since conception the two brothers are compound heterozygous for two class I splice site mutations that target the same exon. Thus, the spontaneous rescue of some functional CFTR activity from two class I mutations is not unlikely: we have investigated CFTR biomarkers in a singular case of a compound heterozygous patient for two non-sense mutations who demonstrated CFTR activity in the ICM (Tümmler, 2019). This index case taught us that not all carriers of two class I mutations lack CFTR activity.

This showcase illustrates that although high-throughput screening of *CFTR* mutations in recombinant cell lines allows proper classification of mutation phenotypes, and standard cell culture assays can predict rare *CFTR* mutation response to highly effective CFTR modulation, the outcome is not necessarily predictive of individual patient response to CFTR modulators *in vivo*. In summary, we provided evidence that some patients with class 1 *CFTR* mutations may benefit from ETI. Hence, when it comes to the issue whether rare or ultra-rare mutations will be responsive to CFTR modulators like ETI, the individual subject should be assessed *in vivo* with CFTR biomarkers prior and during treatment with the medication.

## Data Availability

The original contributions presented in the study are included in the article/Supplementary Material, further inquiries can be directed to the corresponding author.

## Abbreviations

A, adenine; CF, cystic fibrosis; CFTR, cystic fibrosis transmembrane conductance regulator; ELX/TEZ/IVA, ETI, elexacaftor/tezacaftor/ivacaftor; G, guanine; ICM, intestinal current measurement; LCI_2.5_, lung clearance index 2.5; MBW, multiple breath washout; nPD, nasal transepithelial potential difference measurement; QPIT, quantitative pilocarpine iontophoresis; PI-CF, pancreatic insufficient cystic fibrosis; pwCF, people with cystic fibrosis; SST, sweat secretion test.
